# Oligosaccharides Might Contribute to the Antidiabetic Effect of Honey: A Review of the Literature

**DOI:** 10.3390/molecules17010248

**Published:** 2011-12-28

**Authors:** Omotayo O. Erejuwa, Siti A. Sulaiman, Mohd S. Ab Wahab

**Affiliations:** Department of Pharmacology, School of Medical Sciences, Universiti Sains Malaysia, 16150 Kubang Kerian, Kelantan, Malaysia; Email: sbsamrah@kb.usm.my (S.A.S.); msuhaimi@kb.usm.my (M.S.A.W.)

**Keywords:** prebiotics, oligosaccharides, honey, hypoglycemic, anti-diabetic, microbiota, body weight, food intake

## Abstract

Evidence shows that honey improves glycemic control in diabetes mellitus. Besides its hypoglycemic effect, studies indicate that honey ameliorates lipid abnormalities in rats and humans with diabetes. The majority of these studies do not examine the mechanisms by which honey ameliorates glycemic and/or lipid derangements. The gut microbiota is now recognized for its ability to increase energy harvest from the diet and alter lipid metabolism of the host. Recently available data implicate a causal role of these gut microbes in the pathophysiology of obesity, insulin resistance, and diabetes mellitus. In this review, we present some of the latest findings linking gut microbiota to pathogenesis of obesity, insulin resistance, and diabetes mellitus. The review also underlines data that demonstrate the beneficial effects of oligosaccharides on various abnormalities commonly associated with these disorders. Based on the similarities of some of these findings with those of honey, together with the evidence that honey contains oligosaccharides, we hypothesize that oligosaccharides present in honey might contribute to the antidiabetic and other health-related beneficial effects of honey. We anticipate that the possibility of oligosaccharides in honey contributing to the antidiabetic and other health-related effects of honey will stimulate a renewed research interest in this field.

## 1. Introduction

In the past few years, research focus has shifted from the mere pathophysiological role of gut pathogens to the potential beneficial effects of gut microbiota [1]. Microbiota refers to a group of microorganisms (bacteria, viruses, archaea and unicellular eukaryotes) which live as commensals in their hosts [1]. The gut microbiota plays a vital role in host metabolism by enhancing energy harvest from the diet, altering lipid metabolism, modifying endocrine function as well as activating inflammatory tone [2,3]. These microbes are actively involved in the processing, storage, and oxidation of dietary oligosaccharides, polysaccharides, and macronutrients [2]. In the human gut, it is reported that this microbiota consists of a large number and several arrays of microorganisms which comprise at least 10^14^ bacteria [4,5]. Some of the bacterial phyla commonly found in human and mouse gut include Bacteroidetes, Firmicutes and Actinobacteria [5]. Even though the main groups of microbiota can be found in every individual, evidence suggests that there are distinct differences at the species level between different persons [6,7]. A host’s factors such as health, genetic background, diet, sex, age, immune system, as well as intestinal-related factors (pH, peristalsis, mucin secretions, *etc.*), are known to influence and modulate the composition of gut microbiota [8,9,10,11,12,13,14]. Several lines of evidence implicate a causal role of gut microbiota in the pathophysiology of obesity, insulin resistance and diabetes mellitus [5,15,16,17,18].

In both animal and human studies, compelling evidence indicates that honey exhibits hypoglycemic and antidiabetic effects [19,20,21,22]. However, the mechanisms of its hypoglycemic/antidiabetic effect are unknown. Honey, a natural substance produced by bees, comprises predominantly monosaccharides (fructose and glucose) which are absorbed easily in the small intestine [23]. Besides, honey contains oligosaccharides and polysaccharides which are not easily digested and absorbed in the small intestine but large intestine [23,24,25,26]. Oligosaccharides and polysaccharides, which are also found in plants such as onion, chicory, and garlic, are resistant to gastric acidity and hydrolysis by human gastrointestinal digestive enzymes [6,27,28]. They are rich sources of nutrients for intestinal microflora [6,27,28]. Oligosaccharides are regarded as prebiotics [29,30]. Prebiotics are defined as “non-digestible dietary ingredients that beneﬁcially affect the host by selectively stimulating the growth and/or activity of one or a limited number of bacteria in the colon, thus improving host health” [29]. In the most recently updated definition, “a dietary prebiotic is a selectively fermented ingredient that results in specific changes in the composition and/or activity of the gastrointestinal microbiota, thus conferring benefit(s) upon host health” [30].

Fructooligosaccharides, galactooligosaccharides, and lactulose are among the commonly researched prebiotics [28]. Xylooligosaccharides and isomaltooligosaccharides are other oligosaccharides which have been evaluated for their prebiotic effect [29,31,32]. Oligosaccharides and certain food substances (for instance, meals low in fat and high in fruits and vegetables) have been reported to decrease the incidence of chronic diseases such as insulin resistance, diabetes mellitus, hypertension, and metabolic syndrome [33,34,35,36,37,38]. This review presents latest findings that implicate a possible role of gut microbiota in the pathophysiology of obesity, insulin resistance, and diabetes mellitus. It also highlights data that demonstrate the beneficial effects of oligosaccharides in ameliorating obesity, insulin resistance, and diabetes mellitus. Considering the similarities of these compelling findings with those of honey, we hypothesize that oligosaccharides present in honey might contribute to the antidiabetic and other health-related beneficial effects of honey. It is anticipated that the possibility of oligosaccharides in honey contributing to the antidiabetic and other health-related effects of honey will stimulate a renewed research interest in this field. This will help to extend the frontiers of this field and add to the existing literature.

## 2. Evidence Implicating a Potential Role of Gut Microbiota in the Pathogenesis of Insulin Resistance and Diabetes Mellitus

Findings from a number of studies have implicated a role of gut microorganisms in the pathophysiology of insulin resistance and diabetes mellitus. A study by Backhed *et al.* provides the first evidence in support of a causal role of gut microflora in the pathogenesis of obesity and insulin resistance [39]. The study indicated that total body fat (42%) and epididymal fat pad weights (47%) were considerably less (despite higher food intake) in germ-free mice than in conventionally raised mice (with microbiota) [39]. The authors reported that C57BL/6 mice (germ-free model) conventionalized with microbiota harvested from the cecum or distal intestine of conventionally raised mice had a 57% and 61% increase in total body fat and epididymal fat pad weights, respectively. Microbiota also increased lipoprotein lipase activity in epididymal fat pads [39]. The study also revealed that conventionalized mice had elevated levels of fasting glucose, insulin, and leptin as well as insulin resistance [39]. The same study also showed that microbiota enhanced the absorption of monosaccharides and suppressed fasting-induced adipocyte factor in the intestine. Triglyceride synthesis, mRNA expression levels of acetyl-CoA carboxylase, fatty acid synthase activity, sterol response element binding protein 1 (SREBP-1) and to a lesser extent, carbohydrate response element binding protein (ChREBP) were increased in the liver of mice conventionalized with microbiota [39].

Similarly, in a follow up study, Backhed and co-workers reported that germ-free mice fed a high-fat, high-carbohydrate Western diet for two months gained markedly less body weight and fat content than did conventionalized mice [40]. In addition to some of the findings reported earlier, the study found that the density of small intestinal villi capillaries was doubled in conventionalized mice. The authors also found that key pathways (AMP-activated protein kinase and peroxisomal proliferator-activated receptor coactivator-1α) involved in hepatic and muscle fatty acid oxidation were activated [40]. The study further showed that germ-free mice were protected against impaired glucose tolerance and insulin resistance induced by high-fat, high-carbohydrate diet [40]. A study by Turnbaugh *et al.* showed that lean germ-free mice that received microbiota transplantation from diet-induced obese mice had greater fat deposition than lean germ-free mice transplanted with microbiota ecology from lean donors [41]. In germ-free mice transplanted with human fecal microbiota, it was reported that switching from a low-fat, plant polysaccharide-enriched diet to a high-fat, high-sugar Western diet altered the structure, representation of metabolic pathways, and gene expression of microbiota [42]. These data and findings from many other studies, thus, suggest that the type of diet consumed is very crucial to the microbiota ecosystem which may improve or exacerbate insulin resistance and diabetes mellitus [10,39,40,41,42,43,44,45].

Another study by Membrez *et al.* [46] also provides evidence in support of the possible role of gut microflora in contributing to insulin resistance and diabetes mellitus. The authors reported that a combination of antibiotics, norfloxacin and ampicillin, suppressed the numbers of cecal bacteria in ob/ob mice [46]. The study found a significant improvement in fasting blood glucose and oral glucose tolerance following treatment of ob/ob, diet-induced obese and insulin-resistant mice with antibiotic combination. It was also reported that the treated mice had reduced liver triglycerides and increased liver glycogen content [46]. Another independent study also reported similar findings [47]. Cani and co-workers showed that antibiotic therapy improved glucose tolerance, reduced insulin resistance and obesity in high-fat, diet-induced obesity and diabetes in mice [47]. A study by Dumas *et al.* also provides evidence that implicates the role of gut microbiota in the development of insulin resistance [48]. Some studies also suggest that microbiota can activate or aggravate the inflammatory processes of obesity and insulin resistance [47,49].

The evidence which suggests that the gut microbiota can be inherited also supports the potential role of microflora in insulin resistance, obesity, and diabetes mellitus [50]. In Tlr5-deficient and non-obese diabetic mutant mice, which are prone to develop obesity or type 1 diabetes mellitus, evidence has shown that the gut microbiota’s characteristics do influence and contribute to the phenotype of these disorders in the host [5,17,51]. Children with normal weight have been reported to have higher fecal bifidobacterial contents than those becoming overweight [9]. In contrast, in children becoming overweight, a greater number of *Staphylococcus aureus* was reported than in children with normal weight [9]. Similarly, compared with non-diabetic individuals, type 2 diabetic patients have reduced number of Bifidobacteria [52]. A recent study indicated that germ-free mice fed high fat diet had increased insulin sensitivity, improved glucose tolerance, reduced fasting and non-fasting insulin levels. The study also showed that germ-free mice fed high fat diet had reduced plasma TNF-alpha, total serum amyloid A concentrations, and reduced hypercholesterolemia [53]. Germ-free mice were also reported to be associated with reduced expression of Peptide YY [54].

By and large, these data suggest that not only is microbiota capable of increasing energy-harvesting efficiency in the intestine, but can also increase energy storage and reduce energy expenditure [15,39,40]. It is postulated that while this may be beneficial in ancient humans who had variable access to food, it may be detrimental in individuals living in modern and developed societies, where the bulk of diets is calorie-enriched [39]. Thus, it is suggested that changes in microbial ecology, as a results of Western diets and/or differences in microbial ecosystem among individuals residing in these societies, may constitute an important environmental factor that influences or contributes to excess calorie and ultimately enhancing insulin resistance, obesity, and diabetes mellitus [15,39]. Besides, evidence indicates that gut microbiota can contribute to obesity, insulin resistance and diabetes mellitus by promoting synthesis and deposition of lipid, inducing inflammation and through increased secretion of gut-derived peptides [15,39,40,46,47,49,53,54].

## 3. Effect of Oligosaccharides in the Gut and on Gut Microbiota

The oligosaccharides are not metabolized by human intestinal hydrolases/digestive enzymes [55]. Instead, they are hydrolyzed, by gut microbiota, to produce short-chain fatty acids (acetate, lactate, propionate and butyrate), gases (CO_2_, H_2_, and CH_4_) and other metabolites [27,55,56,57]. Factors such as type of sugar monomer, the nature of the glycosidic bonds between sugar moieties, and extent of polymerization influence fermentation of oligosaccharides and other prebiotics in the gut [58]. The ability of gut microbiota to selectively metabolize oligosaccharides is attributed to their intrinsically expressed hydrolytic enzymes such as α-galactosidases, β-galactosidases, β-fructosidase, α-glucosidases, β-glucosidases, and xylanase [55,59,60]. Recent evidence indicates that oligosaccharides are capable of re-establishing the equilibration of gut microbiota ecosystem. Studies have shown that oligosaccharides can suppress the growth and activity of harmful or pathogenic bacteria such as Clostridium, Staphylococcus, Veillonella, Proteus, and Escherichia [6,28,29,61]. On the other hand, oligosaccharides enhance the growth and activity of beneficial or non-pathogenic bacteria such as Bifidobacterium, Lactobacillus, Eubacterium, and some Streptococcus, Enterococcus, and Bacteroides [6,28,29,61].

Human subjects who ingested fructooligosaccharides were reported to have increased fecal bifidobacterial counts and beta-fructosidase activity [62,63]. A recent study, in human flora-associated piglets, showed that short-chain fructooligosaccharides (scFOS) stimulated the growth of beneficial bacteria, Bifidobacteria and Bacteroides, while FOS suppressed the growth of pathogenic Clostridium leptum subgroup [64]. The study found that development or growth of the piglets affected the prebiotic effect of scFOS on these microorganisms except the Bifidobacteria [64]. Another recently published data indicated that FOS supplementation in human subjects significantly increased fecal counts and daily fecal output of bifidobacteria [65]. The study further showed that the effect of FOS on bifidobacteria output was sustained after the discontinuation of FOS supplementation [65]. The enhanced activity or growth of microbiota following oligosaccharide supplementation is ascribed to the oligosaccharide-derived metabolites especially the short-chain fatty acids [58,61]. Evidence also indicates that the short-chain fatty acids are rapidly absorbed in the large intestine, and are subsequently metabolized in certain tissues such as liver and muscle [61,66]. The short-chain fatty acids generated from hydrolysis of oligosaccharides play an important role in the beneficial and systemic effects of oligosaccharides [5,54,61,67,68,69]. Evidence also indicates that FOS enhance the intestinal absorption of mineral ions in mice [70].

## 4. Effect of Oligosaccharides on Elevated Levels of Blood Glucose and Glycosylated Hemoglobin

In rats treated with oligofructose, postprandial glucose was markedly reduced [71,72]. Similarly, improved glucose tolerance was reported in streptpzotocin (STZ)-induced diabetic rats treated with oligofructose [73]. In mice fed high fat, carbohydrate-free diets, administration of fructooligosaccharide resulted in reduced hyperglycemia [74]. In addition, inulin treatment was demonstrated to markedly reduce blood glucose in STZ-induced diabetic rats [75]. Cani and co-workers also showed that oligosaccharide administration lowered glucose-stimulated insulin secretion, improved glucose tolerance, reduced hyperglycemia, and insulin-sensitive hepatic glucose production in high-fat-fed diabetic mice [76]. As documented in several studies, the beneficial effect of oligofructose on elevated blood glucose is mediated partly via increased glucagon-like peptide-1 (GLP-1) secretion [71,73,76,77]. Recent findings also indicate that prebiotic modulation of intestinal microflora improved metabolic impairments, plasma and hepatic inflammation during obesity and diabetes via increased glucagon-like peptide-2 (GLP-2) [78].

Prebiotics ingestion in human subjects resulted in reduced postprandial plasma glucose responses [79]. Overweight and obese individuals supplemented with oligofructose had markedly reduced blood glucose concentrations [80]. Sasaki and colleagues reported that supplementation with transglucosidase, an enzyme that hydrolyzes starch to oligosaccharides, significantly reduced the postprandial blood glucose concentrations in human subjects with impaired glucose tolerance [81]. Similarly, following prebiotic ingestion, reduced postprandial serum glucose was reported in persons with impaired glucose tolerance [82]. FOS supplementation in diabetic subjects significantly reduced fasting blood glucose [83]. In type 2 diabetic subjects treated with arabinoxylan, a prebiotic, hyperglycemia was markedly reduced [84]. Similarly, ingestion of prebiotic soluble fiber, consisting of oligofructose and polydextrose, in type 2 diabetic patients resulted in a significant decrease in glycosylated hemoglobin as well as fasting and postprandial blood glucose concentrations [85]. However, some studies did not find a significant difference in basal hepatic glucose production, insulin suppression of hepatic glucose production, insulin-induced glucose uptake or fasting plasma glucose in healthy subjects or type 2 diabetic patients [86,87]. Other authors also reported no significant effect of dietary inulin or oligofructose on serum glucose levels in hypercholesterolemic or diabetic subjects [88,89].

## 5. Effect of Oligosaccharides on Pancreas and Glucose-Regulating Hormones

Oligofructose was shown to reduce serum insulin level in rats [71]. A study found that fructose-fed rats had increased plasma insulin level and supplementation with oligofructose restored the plasma insulin level similar to that of control [72]. In STZ-induced diabetic rats, oligofructose increased portal and pancreatic insulin concentrations [73]. Eight weeks-administration of inulin to human subjects resulted in reduced fasting insulin level [90]. Short-chain FOS were reported to reduce postprandial insulin response in subjects with mild hypercholesterolaemia [91]. Similarly, type 2 diabetic subjects or individuals with impaired glucose tolerance treated with arabinoxylan had reduced insulin levels [82,84]. A combination of oligofructose and polydextrose was reported to increase insulin and C-peptide levels in type-2 diabetic patients [85]. Similarly, oligofructose was reported to improve glucose-stimulated insulin secretion [76]. However, some studies did not find any significant difference in insulin concentrations or erythrocyte insulin binding in healthy subjects or type 2 diabetic patients supplemented with short-chain oligofructose [86,87].

## 6. Effect of Oligosaccharides on Appetite-Regulating Hormones

Certain hormones such as glucagon-like peptide-1 (GLP-1) and peptide YY are secreted into the gut after food ingestion [92]. They are recognized for their role in glucose homeostasis [93]. Some of their effects include promoting satiety, reducing the rate of glucose absorption, suppressing preprandial glucagon, and attenuating glycemic response to a meal [92,93,94]. They also exert a stimulatory role in pancreas resulting in enhanced insulin secretion and ß-cell proliferation, thus contributing to improved glucose disposal [92,93,94,95]. Other hormones such as gastric inhibitory polypeptide (GIP), leptin and ghrelin are also secreted in response to food ingestion. Oligofructose supplementation in rats increased cecal GLP-1 content and serum GIP concentration [71]. FOS were reported to increase colonic and portal plasma GLP-1 in mice fed high-fat, carbohydrate-free diets [74]. The proximal mucosa of colon of rats fed fructans had increased concentrations of GLP-1 and proglucagon mRNA compared to those of control rats [96]. The oligofructose-fed rats had increased proglucagon mRNA in the cecum and the colon [97]. Similarly, addition of oligofructose in the diet markedly increased the levels of GLP-1 and GLP-2 in the proximal colon, resulting in increased portal concentration of GLP-1 [97]. A study showed that oligofructose treatment in rats doubled the number of enteroendocrine L-cells and GLP-1-expressing cells in the proximal colon [98]. Evidence also showed that prebiotic modulation of intestinal microflora improved plasma and hepatic inflammation and metabolic impairments during obesity and diabetes via increased glucagon-like peptide-2 (GLP-2) [78].

An increase in proglucagon mRNA and peptide content has been reported in the proximal colon of rats supplemented with oligofructose [98]. Oligofructose has been shown to reduce plasma level of active ghrelin in rats [96]. Likewise, in rats fed high-fat diet, addition of oligofructose to the diet resulted in reduced ghrelin level [97]. Oligofructose was also shown to reduce the level of leptin and increase proglucagon and peptide YY gene expression in rodents [72,99]. In patients with gastroesophageal reflux disease, FOS supplementation was associated with markedly increased integrated plasma response of GLP-1 [100]. Reduced total ghrelin response following consumption of arabinoxylan, a prebiotic, was reported in subjects with impaired glucose tolerance [82]. In humans, prebiotic treatment was reported to increase plasma levels of GLP-1 and peptide YY [79]. In overweight and obese adults, oligofructose supplementation was associated with a reduced area under the curve (AUC) for ghrelin and an increased AUC for peptide YY [80].

## 7. Effect of Oligosaccharides on Lipid Metabolism

Administration of oligofructose or inulin to rats significantly reduced the serum and hepatic levels of triglycerides (TG), phospholipids and cholesterol while the high density lipoproteins (HDL) cholesterol / low density lipoproteins (LDL) cholesterol ratio was increased [68,72,96,101]. FOS-fed rats were reported to exhibit reduced epidydimal fat mass, reduced serum TG levels, increased fecal excretions of neutral sterol and volatile fatty acids [102]. Short-chain oligosaccharide supplementation in insulin-resistant rats resulted in reduced hepatic fatty acid synthase activity, plasma TG and free fatty acids (FFA) [103]. In STZ-induced diabetic rats, inulin treatment markedly reduced TG, total cholesterol (TC), LDL cholesterol and atherogenic index while it increased HDL cholesterol [75]. Similarly, amelioration of lipid abnormalities was also reported in obese Zucker rats and high-fat diet-fed rats supplemented with oligofructose [97,104,105]. The beneficial effect of oligosaccharides on lipid metabolism is mediated via reduced hepatic *de novo* lipogenesis [101,106,107]. Another recent study also showed that inulin-type fructans reduced an accumulation of adipocytes, blunted peroxisome proliferator activated receptor gamma (PPARgamma)-activated differentiation factors and caused reduced G protein-coupled receptor 43 (GPR43) expression in the subcutaneous adipose tissue in high-fat diet-fed mice [108]. The study further showed that these effects were mediated via modulation of the gut microbiota, resulting in increased Bifidobacteria and suppressed Roseburia and Clostridium [108]. Oligofructose was shown to reduce alanine aminotransferase (ALT) activity in rats with endotoxic shock induced by lipopolysaccharide (LPS) injection [109].

In diabetic subjects, improved lipid metabolism as evidenced by reduced serum concentrations of TC and LDLcholesterol was reported following FOS ingestion [83]. Inulin treatment markedly reduced levels of triacylglycerol, TC and LDL cholesterol in healthy subjects compared with the levels in controls [90,110]. In men with hypercholesterolemia, inulin significantly reduced serum TG and tended to decrease serum cholesterol [111]. Similar beneficial effects of inulin on lipid metabolism were also reported in human subjects with or without hypercholesterolemia [89,112]. Type 2 diabetic patients administered prebiotics, oligofructose and polydextrose, had markedly reduced TC, LDL cholesterol, TC/HDL cholesterol and LDL cholesterol/HDL cholesterol ratios, TG, VLDL cholesterol and lipoprotein a while HDL cholesterol was increased in these patients [85]. A recent study, conducted in human subjects, showed that increased fecal bifidobacteria output, as a result of FOS supplementation, was associated with reduced plasma cholesterol or increased cholesterol excretion [63,65]. However, some studies did not find any significant difference in fasting parameters of plasma levels of TG, FFA, TC, LDL cholesterol, VLDL cholesterol and HDL cholesterol as well as concentrations of lipoprotein a, apolipoproteins A1 and B following a moderate intake of FOS in healthy individuals, mild hypercholesterolemic or type 2 diabetic patients [86,87,88,91].

## 8. Effect of Oligosaccharides on Body Weight and Food/Energy Intake

FOS or fructans reduced body weight gain and energy intake in rats [96,102]. Administration of FOS to mice fed high-fat carbohydrate-free diets markedly reduced body weight gain and energy intake [74]. In rats fed high-fat diet, oligofructose feeding markedly reduced energy intake and body weight gain [97]. Inulin or oligofructose supplementation significantly reduced food intake in STZ-induced diabetic rats [73,75]. Similarly, oligofructose reduced body weight gain in mice [76]. Other studies have documented the effects of oligofructose on food consumption, energy intake, and body weight in rodents [77]. In humans, prebiotic treatment was reported to reduce hunger rates [79]. Oligofructose ingestion considerably increased satiety after breakfast and dinner while it reduced hunger and food intake following dinner in human subjects [113]. Reduced body weight after oligofructose supplementation in overweight and obese adults was also reported [80]. However, some studies did not find any significant effect of oligosaccharides on body weight and food intake [87,114].

## 9. Documented Effects of Honey Which Are Comparable to Those of Oligosaccharides

Similar to effect of oligofructose or FOS on the growth and/or activity of microbiota [6,28,29,61], a study reported that honey enhanced the growth of five different species of human intestinal Bifidobacteria [115]. The authors found that the prebiotic effect of honey was comparable to those of FOS, galactooligosaccharides, and inulin [115]. Similarly, Sanz and co-workers found that honey oligosaccharides produced prebiotic activity, as evidenced by increased populations of bifidobacteria and lactobacilli, although the prebiotic effect was less compared to that of FOS [25]. Other authors also reported that honey supplementation increased the number of beneficial colonic bifidobacteria and lactobacilli [116]. However, a study that investigated the effect of addition of acacia honey to yogurt milk on the survival of yogurt’s microbes during storage at 4 °C for six weeks found no significant effect of honey on the production levels of lactic acid and viability of *Streptococcus thermophilus* and *Lactobacillus delbrueckii* [117].

As reported for oligosaccharides [73,74,76,79,80,83], honey supplementation attenuated postprandial glycemic response in healthy volunteers and type 1 diabetic patients [118]. Similar reductions in blood glucose were reported in subjects with impaired glucose tolerance [119]. In diabetic rats, honey administration also reduced hyperglycemia [21,22,120,121,122,123,124,125]. Similar to the effect of oligosaccharides on insulin [90,91], treatment of diabetic rats with honey increased serum insulin and improved beta-cell mass [22,125]. Likewise, as documented for oligosaccharides [86,87,88,89], some studies did not find a significant effect of honey on the levels of blood glucose or insulin in diabetic patients [126,127,128]. A study by Bahrami and co-workers found that honey supplementation in type 2 diabetic patients did not produce a significant effect on fasting blood glucose but increased glycosylated hemoglobin [129]. Comparable to the effect of oligosaccharides on leptin, ghrelin, and peptide YY [72,79,80,96,97,99], evidence showed that honey enhanced total peptide YY response and delayed postprandial ghrelin response in healthy nonobese women [130].

Similar to the effect of oligosaccharides on lipid metabolism [72,83,101,104], honey supplementation considerably reduced TC, LDL cholesterol and TG, while it increased HDL cholesterol in type 2 diabetic patients or healthy individuals [19,129,131]. Following honey supplementation, reduced body weight gain or food/energy intake was also reported in healthy rats [128] and in type 2 diabetic subjects [129]. In individuals who are overweight or obese, honey administration mildly decreased or did not increase body weight [131]. Similar weight loss or reduced food intake was reported as a result of oligofructose consumption in healthy, overweight or obese individuals [74,76,80,113]. Similar to the potential hepatoprotective effect of oligosaccharides [109], honey supplementation also produced hepatoprotective effect in STZ-induced diabetic rats [132]. Ezz El-Arab *et al.* also reported that honey administration ameliorated aflatoxin-induced histopathological changes in mice [116]. Intravenous honey also elicited a protective effect against carbon tetrachloride-induced hepatic damage [133].

## 10. Conclusions and Future Perspective

Taken together, the data presented in this review strengthen a potential role of gut microbiota in the pathogenesis of insulin resistance, diabetes mellitus and obesity. The review also underscores numerous studies which demonstrate that oligosaccharides elicit antidiabetic, antilipidemic, and other beneficial effects - via modulation of gut microflora ecosystem. Considering that honey is enriched in oligosaccharides, we hypothesize that the antidiabetic and some other beneficial effects of honey might depend partly on oligofructose present in honey. This is in view of the similarities of findings on the effects of oligofructose and honey on the gut microbiota, metabolism of glucose and lipids, glucose- and insulin-regulating hormones as well as on body weight and food intake. Considering the dearth of data, especially in regard to randomized controlled clinical studies, we believe that future research on honey should not be restricted or limited to investigating the potential beneficial/therapeutic effects alone but should entail studies aimed at elucidating the complex mechanisms of action of honey. It is hoped that this review will stimulate fundamental research aimed at elucidating the mechanisms by which honey improves glycemic control or exerts antidiabetic effect. Studies in both animals and humans are needed to verify or substantiate some of the issues highlighted in this paper. This will help to further extend the frontiers of knowledge as regards health-benefits of honey.
